# Amyloid and tau burden relate to longitudinal changes in the performance of complex everyday activities among cognitively unimpaired older adults: results from the performance-based Harvard Automated Phone Task

**DOI:** 10.3389/fnagi.2024.1420290

**Published:** 2024-06-12

**Authors:** Mark A. Dubbelman, Ibai Diez, Christopher Gonzalez, Rebecca E. Amariglio, J. Alex Becker, Jasmeer P. Chhatwal, Jennifer R. Gatchel, Keith A. Johnson, Joseph J. Locascio, Onyinye J. Udeogu, Sharon Wang, Kathryn V. Papp, Michael J. Properzi, Dorene M. Rentz, Aaron P. Schultz, Reisa A. Sperling, Patrizia Vannini, Gad A. Marshall

**Affiliations:** ^1^Department of Neurology, Massachusetts General Hospital, Harvard Medical School, Boston, MA, United States; ^2^Center for Alzheimer's Research and Treatment, Department of Neurology, Brigham and Women's Hospital, Harvard Medical School, Boston, MA, United States; ^3^Department of Radiology, Massachusetts General Hospital, Harvard Medical School, Boston, MA, United States; ^4^Department of Psychology, Rosalind Franklin University of Medicine and Science, North Chicago, IL, United States; ^5^Department of Psychiatry, Massachusetts General Hospital, Harvard Medical School, Boston, MA, United States; ^6^Division of Geriatric Psychiatry, McLean Hospital, Harvard Medical School, Belmont, MA, United States

**Keywords:** Alzheimer's disease, function, instrumental activities of daily living, amyloid, tau, longitudinal

## Abstract

**Background:**

Changes in everyday functioning constitute a clinically meaningful outcome, even in the early stages of Alzheimer's disease. Performance-based assessments of everyday functioning might help uncover these early changes. We aimed to investigate how changes over time in everyday functioning relate to tau and amyloid in cognitively unimpaired older adults.

**Methods:**

Seventy-six cognitively unimpaired participants (72 ± 6 years old, 61% female) completed multiple Harvard Automated Phone Task (APT) assessments over 2.0 ± 0.9 years. The Harvard APT consists of three tasks, performed through an automated phone system, in which participants refill a prescription (APT-Script), select a new primary care physician (APT-PCP), and transfer money to pay a bill (APT-Bank). Participants underwent Pittsburgh compound-B and flortaucipir positron emission tomography scans at baseline. We computed distribution volume ratios for a cortical amyloid aggregate and standardized uptake volume ratios for medial temporal and neocortical tau regions. In separate linear mixed models, baseline amyloid by time and tau by time interactions were used to predict longitudinal changes in performance on the Harvard APT tasks. Three-way amyloid by tau by time interactions were also investigated. Lastly, we examined associations between tau and change in Harvard APT scores in exploratory voxel-wise whole-brain analyses. All models were adjusted for age, sex, and education.

**Results:**

Amyloid [unstandardized partial regression coefficient estimate (β) = −0.007, 95% confidence interval (95% CI) = (−0.013, −0.001)], and medial temporal tau [β = −0.013, 95% CI = (−0.022, −0.004)] were associated with change over time in years on APT-PCP only, i.e., higher baseline amyloid and higher baseline tau were associated with steeper rate of decline of APT-PCP. Voxel-wise analyses showed widespread associations between tau and change in APT-PCP scores over time.

**Conclusion:**

Even among cognitively unimpaired older adults, changes over time in the performance of cognitively complex everyday activities relate to cortical amyloid and widespread cerebral tau burden at baseline. These findings support the link between Alzheimer's disease pathology and function and highlight the importance of measuring everyday functioning in preclinical disease stages.

## Introduction

The performance of cognitively complex everyday activities, so-called “instrumental activities of daily living” (IADL), changes gradually throughout the Alzheimer's disease (AD) course, eventually leading to a dependence on caregivers for performing such activities as making (doctor's) appointments, managing finances, and using electronic devices. Impairment in IADL is a source of burden, both for the patient and the caregiver (Sherwood et al., 2005; Feger et al., 2020). Yet even before impairment reaches this point, IADL performance is a clinically meaningful outcome. The earliest, subtle difficulties performing IADL are thought to occur at the end of the preclinical phase of AD (Marshall et al., 2012).

The accumulation of cerebral amyloid and the spreading of tau through the temporal lobe characterize early AD (Banerjee et al., 2017; Maass et al., 2018). Tau distribution in the brain appears more closely associated than amyloid distribution with cognitive performance (Lowe et al., 2019; Ossenkoppele et al., 2019). Similarly, IADL functioning has been related to amyloid and tau, both cross-sectionally and longitudinally (Halawa et al., 2019; Marshall et al., 2019b, 2020; Dubbelman et al., 2020, 2022, 2023; Gonzalez et al., 2023). Consequently, it seems that changes in IADL performance might reflect early biological changes associated with AD. Using sensitive instruments is pivotal to uncovering early changes in IADL performance and how they relate to AD pathology.

Assessment of IADL commonly relies on observer and self-reported questionnaires, which attempt to capture self-perceived global everyday functioning. Alternatively, performance-based instruments measure a person's ability to complete one or more specific activities. An example of a performance-based measure is the Harvard Automated Phone Task (APT), which includes several healthcare-related tasks for individuals to perform through a phone menu. Performance on the Harvard APT has been shown to correlate with other measures of IADL (Marshall et al., 2019a), as well as with amyloid and tau cross-sectionally (Gonzalez et al., 2023).

In this study, we aimed to investigate how changes over time in performance on the Harvard APT relate to baseline amyloid and tau. Based on earlier findings with other IADL instruments, we analyzed global cortical amyloid and tau in the medial temporal and neocortical cortices. We hypothesized that, even among those with normal cognition, higher levels of amyloid and tau would be associated with a decreased performance over time on the Harvard APT. Second, in exploratory analyses, we investigated in what (other) areas of the cortex tau deposition related to change over time in IADL performance, to explore regional relationships between tau and everyday functioning.

## Materials and methods

### Participants and procedures

We selected participants from the Instrumental Activities of Daily Living and Subjective Cognitive Decline studies at Massachusetts General Hospital and Brigham and Women's Hospital in Boston, Massachusetts. Participants were included if they were 55 years or older, had an available study partner, and were fluent in English. Exclusion criteria included history of major psychiatric disorder, neurodegenerative disease, brain tumor, or contraindications for magnetic resonance imaging. For the present study, we selected only participants who completed at least two Harvard Automated Phone Task assessments and underwent both amyloid and tau positron emission tomography (PET) scans.

This study was approved by the institutional review board of Partners Healthcare, Inc. All participants provided written informed consent before starting any study procedures.

### Materials

#### IADL assessment

The Harvard Automated Phone Task (APT) was collaboratively developed between the Center for Alzheimer's Research and Treatment of Brigham and Women's Hospital, Massachusetts General Hospital, the Connected Health Innovation at Partners Healthcare, and Rip Road. Harvard APT comprises three tasks in which participants navigate an interactive voice response system: APT-Script, APT-PCP, and APT-Bank, as described in detail by Marshall et al. (2015). Briefly, in the APT-Script task, participants are asked to call their pharmacy to refill a prescription; in the APT-PCP task, participants need to contact their health insurance company to select a new primary care physician; and in APT-Bank, participants make a bank account transfer. Each task consisted of multiple successive steps that the participants needed to complete (e.g., for APT-PCP, enter their member ID, date of birth, select the correct menu items, enter the PCP name and the city). Instructions, including expected responses, were provided to the participants on paper. The number of correct responses divided by the total time to complete each task formed the performance metric for the Harvard APT. Higher values indicate better performance. A detailed description of the Harvard APT and its scoring rules can be found in the first validation paper by Marshall et al. (2015). Participants completed the Harvard APT annually.

#### Tau and amyloid

Positron emission tomography (PET) scans were made for tau, using the [^18^F]-flortaucipir tracer, and amyloid, using the [^11^C]-Pittsburgh compound-B tracer. We computed two aggregate regions of interest (ROIs) for tau-PET: (i) the medial temporal lobe, comprising the amygdala, entorhinal, and parahippocampal regions, and (ii) the temporo-parietal neocortex, comprising the inferior temporal, fusiform, middle temporal, and inferior parietal regions. For amyloid-PET, a large cortical aggregate served as the only ROI. We used FreeSurfer version 5.3.0 to compute binding in the ROIs (Fischl, 2012), represented by the distribution value ratio (DVR) for amyloid or standardized uptake value ratio (SUVR) for tau, with cerebellar gray as the reference region, as described in more detail elsewhere (Chien et al., 2013; Johnson et al., 2016).

In addition to the ROIs, we performed voxel-wise whole-brain analyses to analyze tau binding in relation to change over time in Harvard APT performance. Partial volume correction was applied to all ROI and voxel-wise analyses.

### Statistical analysis

Using linear mixed-effects models (LMMs), we investigated change over time in performance on the Harvard APT tasks, with time in years as the primary fixed effect independent variable and each of the Harvard APT tasks as the primary dependent variable, each in a separate analysis. The random model terms were participant intercepts and slopes of time in years. We used an unstructured covariance structure, allowing any correlation between the random slopes and intercepts to be estimated freely. In the first set of LMMs, we analyzed interactions between time and global cortical amyloid and between time and the two tau-PET ROIs. Three-way interactions between time, tau-PET, and amyloid-PET were also investigated. In the second set of LMMs, we ran the same analyses on voxel-wise data. Voxel-wise analyses were adjusted for multiple testing in MATLAB version 2023a using Monte Carlo simulations. All models were adjusted for baseline age, sex, and education. We report estimates and their corresponding 95% confidence intervals (95% CI), as well as *t*-values (calculated as the estimate divided by the standard error), as an indication of standardized effect size. ROI analyses were run in R version 4.3.2, while voxel-wise analyses were run in R 3.6.3 (R Core Team, 2023).

## Results

Seventy-six participants (aged 72 ± 6 years, 61% female) completed the Harvard APT multiple times. They were either cognitively unimpaired without complaints (CU; *n* = 32, 42%) or had subjective cognitive decline (SCD; *n* = 44, 58%) but had no objective cognitive impairment. Table 1 displays all baseline characteristics. There were no differences in baseline demographic variables between those who were CU or had SCD. The mean follow-up duration was 2.0 ± 0.9 years (range 0.9–3.4 years), with 38 participants (50%) having completed the Harvard APT twice, 28 (37%) having completed it three times, and 10 (13%) having completed it four times. The mean time difference between tau and amyloid PET acquisition and baseline Harvard APT completion was 0.29 ± 0.52 years.

**Table 1:** Baseline characteristics.

**Characteristic**	**Whole group**
*N*	76
Age in years	71.7 ± 5.6
Female, *n* (%)	46 (60.5)
Years of education, *M* (IQR)	18 (15–18)
**Diagnosis**, ***N*** **(%)**	
CU	32 (42.1)
SCD	44 (57.9)
**Race/ethnicity**, ***N*** **(%)**	
Non-Hispanic White	65 (85.5)
Hispanic White	1 (1.3)
Non-Hispanic Black	7 (9.2)
Asian	3 (3.9)
**Cognitive performance**	
CDR sum of boxes	0 (0–0)
MMSE	29.0 ± 1.0
**Harvard APT baseline rate**	
APT-Script	0.09 ± 0.02
APT-PCP	0.04 ± 0.01
APT-Bank	0.04 ± 0.01
**Amyloid**	
DVR	1.35 ± 0.42
Positive status, *n* (%)	16 (21.1)
**Tau SUVr**	
Medial temporal lobe	1.23 ± 0.21
Temporo-parietal neocortex	1.45 ± 0.17
APOE ε4 carrier, *N* (%)	10/49 (20.4)

Data are displayed as mean ± standard deviation, except as noted otherwise.

APOE, apolipoprotein; APT, automated phone task; CDR, clinical dementia rating; CU, cognitively unimpaired; DVR, distribution volume ratio; IQR, interquartile range; M, median; MMSE, Mini-Mental State Examination; SCD, subjective cognitive decline; SUVr, standardized uptake value ratio.

**Figure 1:**
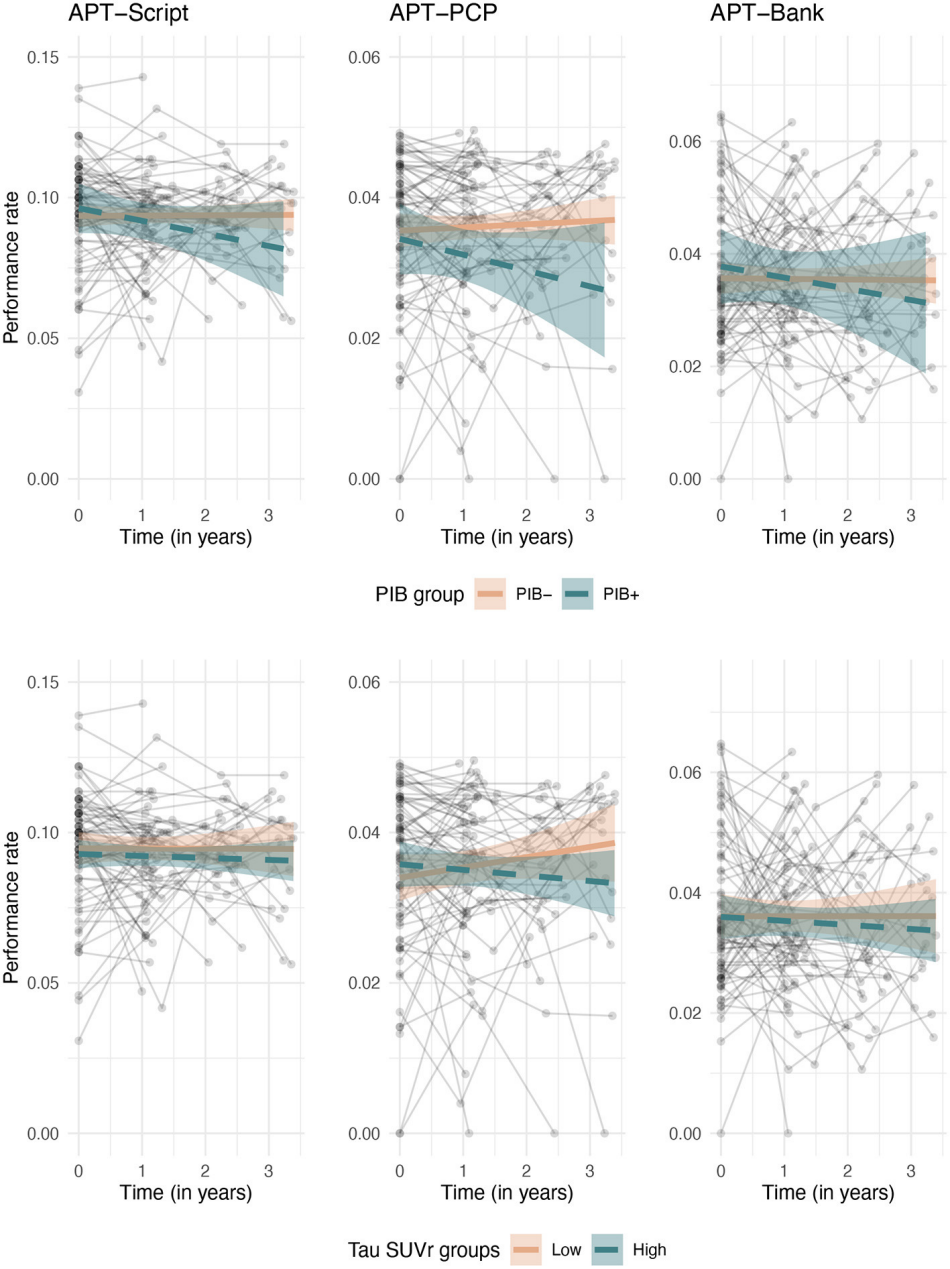
Longitudinal associations between change over time on the Harvard APT tasks and baseline amyloid **(top row)** and medial temporal tau **(bottom row)**. For visualization purposes, amyloid is binarized into amyloid positive and negative groups based on our center's local cutoff, and tau is split into low and high strata based on median split.

In the whole sample, performance on all three Harvard APT tasks was stable over time, i.e., there was no significant change over time. A greater global cortical amyloid burden at baseline was associated with a greater rate of decline in performance over time on APT-PCP [interaction coefficient (β) = −0.003, 95% confidence interval (95% CI) = (−0.006, −0.001), *Z* value (Z) = −2.40], but not on APT-Script or APT-Bank. Greater medial temporal lobe tau was also associated with decreased performance on APT-PCP over time [β = −0.008, 95% CI = (−0.013, −0.004), *Z* = −3.56]. Tau in the temporo-parietal neocortex was not associated with a change in performance on any of the APT tasks. A modest three-way interaction between time, amyloid, and medial temporal tau existed for APT-Script, but not for the other tasks [β_amyloid × *time*_ = −0.055, 95% CI = (−0.103, −0.006), *Z* = −2.22; β_tau × *time*_ = −0.042, 95% CI = (−0.082, −0.001), *Z* = −2.05; β_amyloid × *tau*×*time*_ = 0.031, 95% CI = (0.002, 0.059), *Z* = 2.14]. All results are displayed in Table 2. Figure 1 shows the relationship between amyloid (top row) and medial temporal tau (bottom row) and changes over time on the three APT tasks.

**Table 2:** Longitudinal models of change in Harvard APT tasks, in relation to tau and amyloid.

**Task**	**APT–Script**		**APT–PCP**		**APT–Bank**	
	β **(95% CI)**	***Z***	β **(95% CI)**	***Z***	β **(95% CI)**	***Z***
**Model 1: time only**						
Time	−0.001 (−0.003, 0.001)	−0.83	0.000 (−0.001, 0.001)	−0.27	−0.001 (−0.002, 0.001)	−0.7
**Model 2: time** × **amyloid**						
Time	0.003 (−0.005, 0.010)	0.67	0.004 (0.001, 0.008)	2.32^*^	0.002 (−0.004, 0.007)	0.54
Amyloid	0.001 (−0.012, 0.009)	0.23	−0.000 (−0.006, 0.005)	−0.10	−0.001 (−0.007, 0.006)	−0.16
Time × amyloid	−0.003 (−0.008, 0.003)	−0.96	−0.003 (−0.006, −0.001)	−2.40^*^	−0.002 (−0.006, 0.003)	−0.77
**Model 3a: time** × **MTL tau**						
Time	0.005 (−0.008, 0.018)	0.80	0.010 (0.004, 0.016)	3.47^*^	0.007 (−0.002, 0.016)	1.61
Tau	0.003 (−0.017, 0.023)	0.27	0.006 (−0.004, 0.017)	1.17	0.006 (−0.006, 0.019)	1.04
Time × tau	−0.005 (−0.015, 0.005)	−0.96	−0.008 (−0.013, −0.004)	−3.56^*^	−0.006 (−0.013, 0.001)	−1.75
**Model 3b: time** × **temporo–parietal neocortical tau**						
Time	0.007 (−0.012, 0.027)	0.47	0.008 (−0.002, 0.018)	1.66	0.007 (−0.007, 0.021)	0.95
Tau	0.002 (−0.024, 0.028)	0.17	0.005 (−0.009, 0.019)	0.69	0.001 (−0.015, 0.018)	0.18
Time × tau	−0.006 (−0.020, 0.008)	−0.83	−0.006 (−0.013, 0.001)	−1.71	−0.005 (−0.016, 0.005)	−1.04
**Model 4a: time** × **MTL tau** × **amyloid**						
Time	−0.008 (−0.060, 0.045)	−0.28	−0.001 (−0.027, 0.024)	−0.11	0.013 (−0.027, 0.052)	0.63
Tau	−0.012 (−0.057, 0.081)	−0.34	−0.003 (−0.040, 0.035)	−0.13	0.043 (0.001, 0.085)	2.00^*^
Amyloid	−0.003 (−0.059, 0.066)	−0.10	−0.014 (−0.048, 0.020)	−0.79	0.028 (−0.010, 0.067)	1.45
Time × tau	0.005 (−0.031, 0.041)	0.28	0.000 (−0.017, 0.018)	0.03	−0.011 (−0.038, 0.015)	−0.83
Time × amyloid	0.007 (−0.030, 0.045)	0.39	0.008 (−0.011, 0.026)	0.81	−0.003 (−0.032, 0.026)	−0.20
Time × amyloid × tau	−0.006 (−0.029, 0.018)	−0.46	−0.005 (−0.017, 0.006)	−0.92	0.003 (−0.015, 0.021)	0.32
**Model 4b: time** × **temporo–parietal neocortical tau** × **amyloid**						
Time	0.072 (0.007, 0.136)	2.20^*^	0.024 (−0.009, 0.057)	−1.46	−0.000 (−0.050, 0.050)	−0.01
Tau	0.076 (0.003, 0.148)	2.06^*^	−0.008 (−0.032, 0.048)	−0.38	0.037 (−0.009, 0.083)	1.57
Amyloid	0.091 (0.003, 0.179)	2.03^*^	−0.002 (−0.051, 0.047)	−0.08	0.043 (−0.013, 0.099)	1.52
Time × tau	−0.042 (−0.082, −0.001)	−2.05^*^	−0.013 (−0.033, 0.008)	−1.21	−0.000 (−0.032, 0.031)	−0.03
Time × amyloid	−0.055 (−0.103, −0.006)	−2.22^*^	−0.017 (−0.041, 0.008)	−1.34	0.004 (−0.033, 0.042)	0.23
Time × amyloid × tau	0.031 (0.002, 0.059)	2.14^*^	0.008 (−0.006, 0.022)	1.12	−0.003 (−0.025, 0.019)	−0.26

All models were run separately and were adjusted for baseline age, sex, and years of education. An asterisk (^*^) indicates that the test statistic (t-test) was significant at p <0.05.

APT, automated phone task; MTL, medial temporal lobe; PCP, primary care physician; β, unstandardized partial regression coefficient.

**Figure 2:**
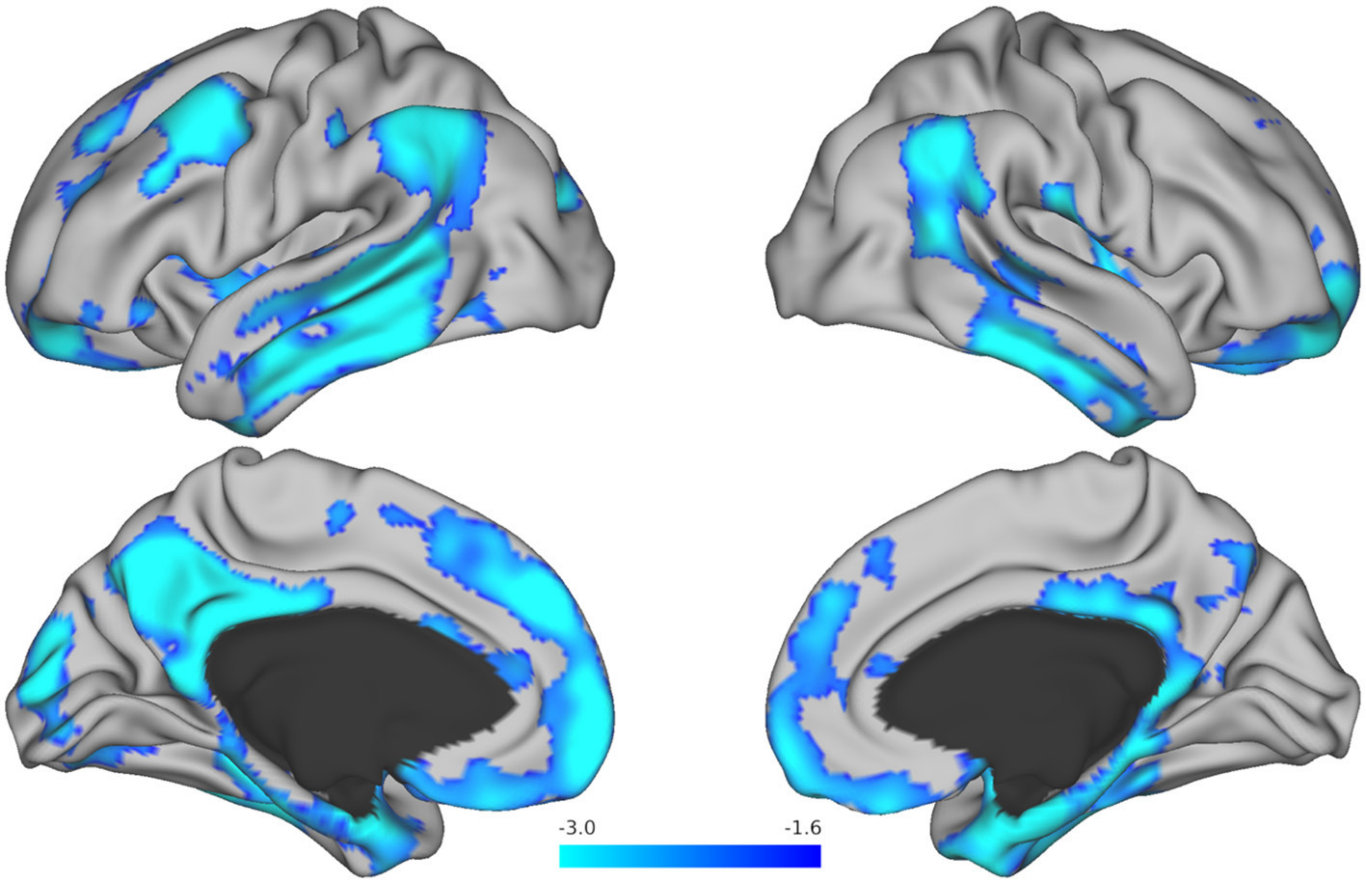
Brain maps resulting from voxel-wise analysis showing tau regions associated with change over time in the Harvard APT-PCP task. The *T*-values for areas that remained significant after correction for multiple comparisons are displayed in blue. Brighter blue colors represent more robust associations.

Next, in exploratory analyses where we were not bound by predefined ROIs, we analyzed how a change in performance on the Harvard APT tasks over time related to baseline tau at the voxel level. Change over time in APT-Script scores showed associations with tau deposition in the left posterior cingulate gyrus, angular gyrus, precuneus, occipital pole, middle temporal gyrus, and temporal fusiform cortex, as well as the bilateral frontal orbital cortex. These associations did not remain significant after adjustment for multiple comparisons. Bilateral tau depositions in the middle and inferior temporal gyrus, supramarginal gyrus, frontal pole, and angular gyrus were associated with changes in performance over time on APT-PCP, including after adjustment for multiple comparisons. Furthermore, tau in the right parahippocampal and left superior and middle frontal and left superior temporal gyri, and left occipital pole was associated with a change in APT-PCP, including after adjustment for multiple comparisons. Figure 2 serves as an illustration and displays brain maps of the areas where tau burden was significantly associated with change over time in the APT-PCP task. Finally, changes over time in APT-Bank scores were associated with left middle frontal gyrus, superior parietal lobule, and temporal fusiform cortex tau, right posterior cingulate and precuneus tau, and bilateral parahippocampal gyrus and thalamus tau. None of these areas remained significant after multiple comparison corrections.

## Discussion

In this study, we found that changes in performance over a follow-up span of 2–4 years on a performance-based measure of IADL, the Harvard APT, related to baseline amyloid and tau deposition among cognitively unimpaired older adults. Change over time on one complex task specifically, where participants needed to select a new primary care physician, showed associations with widespread tau throughout the cortex.

Performance on the three Harvard APT tasks did not change over time at the group level. People without AD commonly show practice effects after retaking a cognitive test (Goldberg et al., 2015), as repeated exposure to a cognitive test leads to improved performance over time. Practice effects were not evident on any of the Harvard APT tasks in this sample, with the average performance remaining stable over annual assessments at the total group level. We did, however, observe a variety of individual trajectories of change over time, with some individuals showing improvement and others showing decline over time. Potential explanations for the absence of an apparent group-level practice effect on the Harvard APT tasks include the assessment frequency (annually), the possibility that individual improvements and decreases in performance cancel each other out, or the design of the Harvard APT, which is not a standard cognitive test but a measure of cognitively complex everyday tasks. It is also possible that the follow-up duration was insufficiently long for detecting group-level changes in cognition, particularly among older adults with minimal AD biomarkers (Soldan et al., 2016; Dubbelman et al., 2024).

Here, we show that a performance-based assessment, administered unsupervised through an automated phone menu, can detect changes in the performance of everyday tasks that are related to underlying Alzheimer's disease processes in individuals who were initially cognitively unimpaired, even over a relatively short follow-up duration. Specifically, the APT-PCP task showed associations with tau throughout the cortex and even in some subcortical structures. A higher tau burden in the temporal, frontal, and occipital lobes appeared to be related to decreased performance over time on APT-PCP. Notably, higher medial temporal tau was linked to decreased performance over time on APT-PCP. This area is known to accumulate tau in relatively early Alzheimer's disease stages (Vogel et al., 2021), and greater temporal tau has been previously associated with cognitive decline (Chen et al., 2021). It thus seems that early tau tracks with early changes in cognitively complex everyday functioning as measured by the Harvard APT.

In addition to the associations with medial temporal tau, a decline in performance on the APT-PCP subtask over time is also related to global cortical amyloid. It has previously been argued that both amyloid and tau abnormalities are required before cognitive decline occurs in the preclinical stages of Alzheimer's disease (Sperling et al., 2019). Our analyses, however, did not demonstrate any interactions between amyloid and tau and the change over time in APT-PCP scores. We did find such interaction, albeit a weak one, in relation to APT-Script scores, where the effects of amyloid and tau on change both individually suggested a declining performance over time, and together show a positive three-way interaction. As a caveat, only approximately a quarter of the participants in our sample were considered to have an amyloid-positive PET scan, meaning that the amount of amyloid accumulation was relatively low. Still, with these minimal levels of amyloid and tau, we did observe significant associations with the Harvard APT. As the change in performance was only apparent in the presence of increased amyloid and tau, the Harvard APT might be a suitable outcome measure for measuring complex everyday functioning among cognitively unimpaired individuals with limited levels of Alzheimer's disease-related pathology.

These findings provide further evidence for the existence of a relationship between everyday functioning and amyloid and tau. Previous studies have demonstrated that the performance of cognitively complex everyday activities is associated with underlying Alzheimer's disease processes, i.e., accumulation of cerebral amyloid and tau (Okonkwo et al., 2010; Marshall et al., 2011, 2019b; Halawa et al., 2019; Dubbelman et al., 2023). This holds true even among cognitively unimpaired individuals, who show no or only limited changes in the performance of everyday activities (Lilamand et al., 2018; Gonzalez et al., 2021; Dubbelman et al., 2022). Thus, it seems that Alzheimer's disease-related changes in everyday functioning emerge in the early stages of the disease trajectory, before the onset of dementia or even mild cognitive impairment (Marshall et al., 2012, 2020; Dubbelman et al., 2020).

Previous work examining the relationship between AD biomarkers and everyday functioning has primarily employed self- or study partner-reported questionnaires, which reflect either the person's own perception—or that of a spouse, friend, or relative—of the difficulties they experience in everyday life. While these questionnaires usually present information about a wide range of activities, they are subject to reporter bias. It has, for instance, been shown that people with more depressive symptoms report more negatively about their own daily functioning (Verrijp et al., 2021). On the other hand, performance-based measures might reflect actual performance more closely (Wesson et al., 2016), albeit in a highly controlled environment and usually focused on a narrower range of activities. Others have previously shown that performance-based assessments of everyday functioning are associated with neurodegeneration among cognitively unimpaired individuals, thus demonstrating the clinical value of performance-based measures in this early disease stage (Keleman et al., 2022). A comparison of self- or study partner-reported questionnaires and performance-based measures of everyday functioning in cognitively unimpaired individuals at risk for Alzheimer's disease might provide more insight into the utility of these different assessments.

The significant associations in the primary analyses using ROIs, as well as the exploratory voxel-wise analyses, were noted with the APT-PCP task but not the APT-Script or APT-Bank tasks. This aligns with prior cross-sectional analyses showing an association between cortical thinning and APT-PCP (Marshall et al., 2015) and between the interaction of amyloid and tau and APT-PCP (Gonzalez et al., 2023). The latter study also showed associations with APT-Bank. APT-Script is a more straightforward task that primarily relates to processing speed, while APT-PCP and APT-Bank are more complex tasks that primarily relate to executive functioning (Marshall et al., 2015). Therefore, the more complex tasks may be more sensitive to early changes in Alzheimer's disease.

We should note that the radioactive tracers used to obtain both amyloid and tau PET scans may bind to proteins or tissues that they are not supposed to bind to; this is referred to as off-target binding, and it is undesirable. Off-target binding is a well-described issue with the flortaucipir tracer, particularly in the basal ganglia, choroid plexus, and other areas (Leuzy et al., 2019). This might explain why we observed a relationship between striatal tau and performance on APT-Script and between the putamen and APT-PCP, which do not appear to bear apparent clinical significance. The findings in the medial temporal cortex are more credible, as they emerged from different analyses as well and were less affected by off-target binding.

Our study had a few limitations. The sample was predominantly non-Hispanic White and highly educated, limiting the generalizability of these results to other populations. Further, the sample was relatively small, and the follow-up duration was relatively short. The results from this study should be interpreted with these limitations in mind. While a larger sample and more extensive follow-up might have shown more or stronger relationships, even with these data we show changes over time in performance in relation to amyloid and tau. Important strengths of this study include the availability of baseline amyloid and tau PET scans, which allow for localization of Alzheimer's disease-related pathology with a high spatial resolution.

In conclusion, our study demonstrated that changes in everyday functioning over time, as measured using a performance-based assessment, are related to baseline amyloid and tau among cognitively unimpaired individuals. As we unravel the early disease processes, self-completed performance-based measures of everyday functioning, such as the Harvard APT, might allow for the detection of early decline in the face of Alzheimer's disease pathology and should be considered as clinically meaningful outcome measures in prevention trials.

## Data availability statement

The raw data supporting the conclusions of this article will be made available by the authors, without undue reservation.

## Ethics statement

The studies involving humans were approved by Partners Healthcare, Inc./Mass General Brigham. The studies were conducted in accordance with the local legislation and institutional requirements. The participants provided their written informed consent to participate in this study.

## Author contributions

MD: Conceptualization, Formal analysis, Investigation, Methodology, Visualization, Writing – original draft, Writing – review & editing. ID: Data curation, Formal analysis, Visualization, Writing – review & editing. CG: Writing – review & editing. RA: Funding acquisition, Writing – review & editing. JB: Resources, Writing – review & editing. JC: Resources, Writing – review & editing. JG: Writing – review & editing. KJ: Writing – review & editing. JL: Writing – review & editing. OU: Data curation, Writing – review & editing. SW: Data curation, Writing – review & editing. KP: Writing – review & editing. MP: Data curation, Writing – review & editing. DR: Writing – review & editing. AS: Writing – review & editing. RS: Writing – review & editing. PV: Resources, Writing – review & editing. GM: Conceptualization, Supervision, Writing – review & editing.
